# Design of an Interactive Nostalgic Amusement Device with User-Friendly Tangible Interfaces for Improving the Health of Older Adults

**DOI:** 10.3390/healthcare8020179

**Published:** 2020-06-19

**Authors:** Chao-Ming Wang, Shih-Mo Tseng, Chen-Siang Huang

**Affiliations:** 1 Department of Digital Media Design, National Yunlin University of Science and Technology, Douliu 64002, Taiwan; jimmy820606@yahoo.com.tw; 2 Graduate School of Design, National Yunlin University of Science and Technology, Douliu 64002, Taiwan; mo0321000@yahoo.com.tw

**Keywords:** older adults, active aging, interactive device, tangible user interface, physical activity, game, sensory stimulation, health promotion

## Abstract

To help older adults achieve active aging, an interactive device with tangible interfaces is proposed, which combines human–machine interaction techniques and older adults’ life experiences to provide three functions: nostalgia, leisure, and entertainment. Firstly, by a review of related theories and studies, principles for designing a desirable interactive device were established. Accordingly, a prototype device with an interactive game was constructed, which was then tested in a field experiment and improved according to the users’ opinions collected by interviews. Subsequently, in a second experiment the users’ and some experts’ feedback about the system’s usability and user interaction satisfaction was collected and analyzed, and found to be reliable and valid for further evaluations of the effectiveness of the proposed system, resulting in the following findings about the older adults who have played the game: the participants become more willing to use technological devices; involving them in the game creates positive aging effects; participants become more receptive to technology products; the participating older adults’ cognitive abilities are trained and their body exercises increased, creating sensory stimulation and health promotion effects; and considering users with cognitive impairments who have stress and difficulty operating the device, the system needs to be improved in the future.

## 1. Introduction

According to the World Health Organization’s (WHO) definition, a person 65 years old or over is said to be an older adult. When a nation’s older population accounts for 7%, 14%, and 20% of its population structure, the nation is called an aging, aged, and super-aged society, respectively [1].

In a 2018 report by the Department of Statistics of the Ministry of the Interior [2], the older people population in Taiwan was 3.31 million, which accounts for 14.1% of the total population indicating that Taiwan has formally entered the stage of an aged society. Also, the aging index, which is defined as the number of people aged 60 and older for every 100 youths younger than 15, reached 107.4 in March 2018, which was the first time that the older people population was larger than the youth population. The aging index is particularly serious in the rural counties in Taiwan. A population-related projection offered by the National Development Council [3] indicated in its final report that Taiwan will enter the stage of a super-aged society soon, and the speed of aging in the population will go faster than developed countries such as Japan and those in Europe and America. Facing the trend of a rapidly growing aging population, complicated and multifaceted problems have arisen. Providing older adults better care services and assisting them to adapt to the aging process has become an important issue and a challenge now.

Rama et al. [4] pointed out that quite a few adults have experienced difficulties in using present-day consumer products with extensive functionalities, and these difficulties came mainly from three factors existing in technology products: (1) complexity of the user interface; (2) age effect in perceptual, cognitive, and motor abilities; and (3) generation-related lack of experience with technology products. Similar observations were also made by Holtzblatt [5] as well as by Chen and Chan [6], pointing out that older adults, due to the decline in their perceptive and cognitive capabilities, often encounter difficulties in using technology products. Consequently, older adults usually regard themselves as incapable of using new technology products and tend to like products which have simple interfaces and can be operated easily.

In addition, with the advance of science, nowadays, technology products are present everywhere, and for older adults it is impossible to avoid facing them in daily lives; on the contrary, appropriate applications of technology products to meet daily living requirements can improve their life quality, creating higher feelings of self-satisfaction [7,8]. Furthermore, it is often emphasized in related studies [9,10,11] that through regular exercises, older adults can promote their own physical fitness, increase their muscle endurance and leg strength, slow down their physical deterioration, and live healthier in an active aging process. Therefore, helping older adults become interested in regular exercises and engaged in it via amusement and recreation has become an important issue.

It is worth mentioning the projects iStoppFalls, FARSEEING, and PreventIT [12,13,14] in Europe which have made good achievements in introducing information and communication technology (ICT) into older adults’ daily lives. The iStoppFalls project [12] was carried out by a research group consisting of scholars from universities in Germany, Austria, Spain, and Holland as well as experts from the Kaasa Solution GmbH and the Neuroscience Research Australia (NeuRA), aiming at helping older adults conduct persistent exercise trainings at home and reduce the danger of falling in their lives. FARSEEING [13] is a collaborative European Commission-funded research project with ten partners distributed in five Europe Union countries aimed at promoting better prediction, identification, and prevention of older adults’ falls with a focus on the use of ICT devices, yielding many innovative R&D results about ICT equipment for the purpose of caring for older people. And the PreventIT project [14] was executed by an interdisciplinary group consisting of researchers from five famous universities, two university hospitals, and two companies with achievements in the aspects of technology development of prevention science, research of medical and behavior science, and solution of communication, creating important influences in the fields of aging and ICT.

Based on the above discussion, the following information was collected in this study:

(1) reviews of studies on the physiological and psychological influences brought on older adults by the hypofunctioning of their perceptual, cognitive, and motor abilities;
(2) understanding of the situation that a country has entered the stage of an aged society; and
(3) the needs of technology-oriented products for older adults.

It was tried in this study to evaluate the effectiveness of improving older adults’ physiological and psychological functions via the use of an interactive device. In particular, the following research problems were investigated in this study:

(1) how to introduce technology into older adults’ lives and design an appropriate interactive device for them to use;
(2) how to design suitable device interfaces for older adults to easily and smoothly learn to use with no stress; and
(3) how to bring positive assistance to older adults through sensory experiences.

More specifically, the aim of this study was to improve older adults’ psychological and physiological states via the use of a specially designed interactive device and help them face aging with positive and happy attitudes to live in LOHAS (lifestyles of health and sustainability); therefore, three aspects of related issues were explored in this study, namely, (1) analysis of usage needs for older adults, (2) design of an interactive device suitable for older adults, and (3) evaluation of sensory experiences gained by using the device, as described in more details in the following.

(1) Analysis of usage needs for older adults—
  We attempted to understand older adults’ physical and mental functions, lifestyles, and perception behaviors from literature reviews and to survey examples of interactive devices for gaining sensory experiences so as to analyze the usage needs for designing an interactive device that is suitable for older adults.
(2) Design of an interactive device for older adults—
  The above analysis results about the usage needs were combined further with three elements of system characteristics, namely, nostalgia, leisure, and entertainment, to design an interactive device that is suitable for older adults.
(3) Evaluation of sensory experiences gained by using the device—
  Interactive environments were set up and the effectiveness of the designed interactive device for older adults to gain sensory experiences was evaluated by the methods of questionnaire surveys and interviews.

## 2. Literature Review

In this section, a survey of related theories and existing studies from various viewpoints about the design of an interactive device for older adults is given, followed by a brief description of the proposed interactive device.

### 2.1. Lifelong Learning Promotion and Active Aging

Aging is the change of a person’s body functions and will expedite the loss of an older adult’s muscle strength, weaken his/her self-care ability, and increase the risk of falling [15,16]. The elements that influence the aging of an older adult include genetics, lifestyle, and disease which, in turn, influence his/her health condition; if he/she can exercise his/her body to keep their organ’s functioning well, it will effectively improve the quality of their lives and decelerate the aging speed [17].

In 2002, the WHO proposed the concept of active aging or participatory aging, saying that if aging is to be a positive experience, longer life must be accompanied by continuing opportunities for health, participation, and security [18]. Also, WHO emphasized the premise of lifelong learning which is one of the central pillars of active aging [19].

Many scholars proposed theories about positive aging: healthy aging, active aging, robust aging, and gerotranscendence, encouraging all older adults to hold positive and aggressive attitudes on facing the aging problem [20,21,22,23,24,25]. For active aging, it will be beneficial to apply ICT to the design of aging-related products for older adults, helping them to decelerate their hypofunctions and satisfy their living and care needs [26].

### 2.2. Tangible User Interfaces

**Figure 1:**
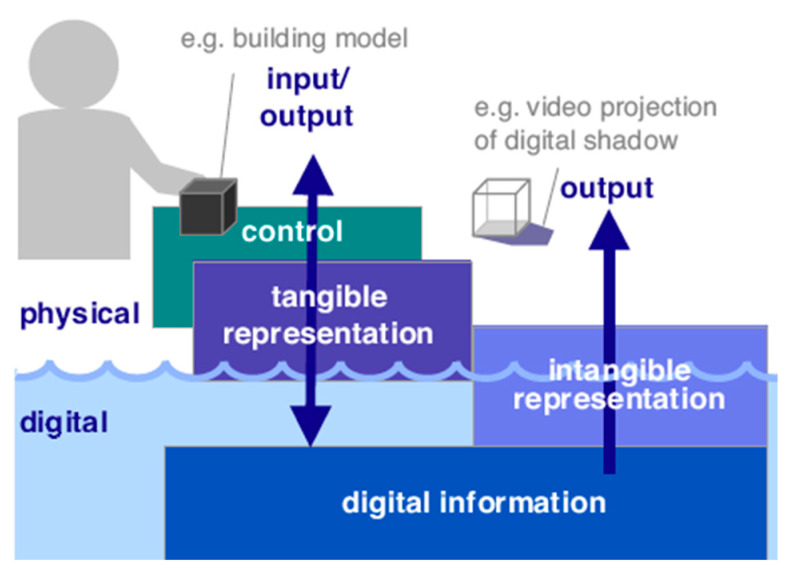
An illustration of the concept of tangible user interface (TUI) [29].

Nielsen [27] proposed a principle to design a good user interface for older adults, i.e., the complexity of software and the system environment should be reduced so that the user can enjoy the operations of interactions in a simple and effective way. Ishii and Ullmer [28] of the Tangible Media Group at the Massachusetts Institute of Technology (MIT) proposed the concept of tangible bit to bridge the gap between the worlds of bits and atoms through a new type of human–computer interface (HUI), called tangible user interface (TUI). As shown in Figure 1, Ishii [29] elaborated the idea by emphasizing the viewpoint of giving physical forms to digital information so as to make the information directly manipulatable with human hands and perceptible through human peripheral senses. The TUI is more intuitive for human beings to use to conduct more effective message communication [30]. In this study, the TUI approach will be followed to design an interactive device with desirable interfaces for older adults.

### 2.3. Sensory Stimuli and Training

In this study, we aimed to explore older adults’ sensory experiences of using the proposed interactive device by checking, after the older adults used the device, whether the effect of sensory stimulus and training can be achieved or not. To this end, at first the definition of sensory perception was investigated which refers to the special organs that accept outside stimuli and the sensory nerves that are spread inside the human body. For older adults, however, their perception capabilities have great differences from when they were young [15].

A person’s sensory experiences can be divided into two levels: physiological and psychological, where the first level refers to various information caused by outside stimuli which is collected by the person’s sensory organs and sent to the brain to form meaningful messages or images as an experience of the second level [31]. In addition, experiences can also be explained as a person’s cognitive feelings obtained from real-life events: the aged are less capable of doing complicated work for which their movements are slower than young people, their reactions are longer, and their eye-tracking actions for web access (i.e., reading) take more time [32,33,34].

### 2.4. Principles for Designing Interactive Devices and Man–Machine Interfaces

In this study, a survey of the principles for designing interactive devices used in existing studies was conducted. Jennifer et al. [35] explained “interaction” as one way of communication which creates cognitions and feedbacks when people on two sides express messages via interactive behaviors. Ijsselsteijn et al. [36] indicated that compared with young people, older adults’ problems in performing their physiological functions are more severe because of their lack of technological knowledge and limited experience with using technology products.

Chen and Chan [6] thought that the main factors for older adults to be willing to use new technology products were ease of use and usefulness. Therefore, the only way to increase their willingness to use a technological product was to design the product based on their needs in daily lives and system operations [37,38]. Specifically, Schieber [39] explored the interface design principle from the aspect of considering older adults’ feelings in order to help them face problems caused by the advance of technology, proposing nine guidelines for designing interfaces involving human vision and hearing functions, respectively, as shown in Table 1.

### 2.5. Survey of Existing Studies and Comparisons

Bong et al. [40] conducted a review regarding studies of TUIs. The results show that very little research has been published, given that the TUI concept was introduced more than 20 years ago. Several recommendations for future research were proposed, including getting older people involved in the process from designing to evaluating the prototype and investigating the effect of the TUI on older adults’ social interactions and health.

“Nostalgia” is a prototype device designed by Nilsson et al. [41] for use by older adults to listen to old news and music with three design features: (1) increasing the older people’s social interaction; (2) bringing an artefact containing information technology into their milieu; and (3) transmitting old news and music from different decades during the twentieth century in a nostalgic way. The interface is a simple “textile runner” unevenly divided into sections representing decades, and pressing a “button” will trigger an old-fashioned radio on a table to play nostalgic music of the corresponding decade. An evaluation with a target group showed that “Nostalgia” could be an appreciated artefact in their everyday lives. However, digital-media and interactive-gaming techniques were not used in the design of the device.

“Aestimo” is a device designed by Rodríguez et al. [42] with a tangible interface composed of a handset, a knob, a book, etc., aimed at evaluating older adults’ user experiences. To play the device, a user wears the handset to listen to voices reading the questions of a questionnaire printed on the book and turns the knob to answer the questions. By wearing the traditional handset and interacting via voices, an older user can feel the kindness and friendliness of the system. An evaluation of the interface in a study with 20 older adults indicated that such an interface is an encouraging introduction to technology and that the physical interaction can activate the senses and the mind. Though “Aestimo” has unique features, it seems to have no visual feedback and less characteristics of digital media and interactive games.

“NikVision” is a tangible tabletop designed by Cerezo et al. [43] based on a user-centered design approach for the cognitive stimulation of older people with cognitive impairments and dementia problems in nursing homes. The general experiences of the users when working with the tangible tabletop was assessed and applied to the design of new cognitive and physical stimulation activities for them. From these experiences, guidelines for the design of tangible activities for this kind of users were extracted for the design and evaluation of tangible activities that could be useful for other researchers.

Based on the results of an evaluation of existing games indicating that exergames have a positive impact on older people, helping them to remain active and contributing to their overall well-being and increased mobility, Planinc et al. [44] developed an exergame named “FishCatcher” for older players which is controlled by body movements. Challenges in applying theoretical guidelines to practical games are discussed using FishCatcher as an example that is helpful to train older adults’ hand movement and hand-eye coordination.

Comparing with the devices mentioned in the above survey, it can be seen that the proposed device has most of the characteristics of the surveyed devices as well as the following unique features: (1) introducing ICT and digital media techniques into the design of a tangible interface for older adults; (2) integrating the audio-visual, brain-cognitive, and hand-moving capabilities of older adults into design the interface; and (3) combining the three elements of nostalgia, leisure, and entertainment into the design of the interactive game of the proposed device.

### 2.6. Brief Description of the Proposed Research

**Figure 2:**
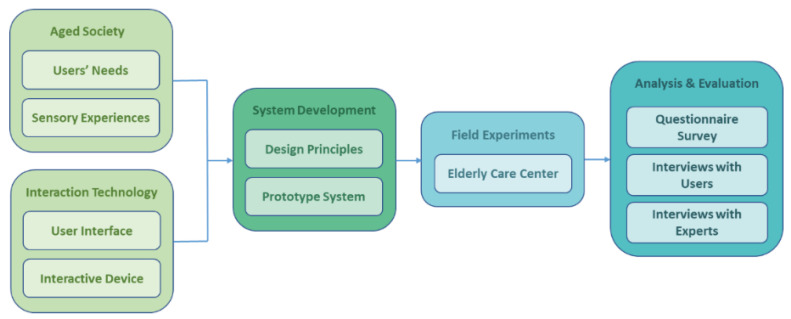
Illustration of the research process of this study.

In this study, as shown in Figure 2, two major issues regarding older adults were investigated, namely, the aged society and interaction technology. Regarding the former issue, the users’ needs and their sensory experiences were collected at the beginning of this study; for the latter, literature reviews of existing studies on interactive devices and related user interfaces were conducted. The results were utilized to induce some design principles based on which a prototype interactive device with tangible interfaces for older adults was constructed. Also built on the proposed device was a game providing the three previously-mentioned functions for older adults, namely, nostalgia, leisure, and entertainment. Two field experiments were then conducted in an older people care center where 7 and 30 older adults were invited to play the game on the prototype device in the two experiments, respectively. The results of the interviews conducted in the first experiment about the participating older users’ feelings and sensory experiences of playing the proposed interactive game were used to improve the prototype device for use in the second experiment. Questionnaire surveys of the users’ comments and collections of opinions from interviews with several experts were conducted in the second experiment, followed by analyses of the data to prove their reliability and validity as well as evaluations of the data from various viewpoints to prove the effectiveness of the proposed device for the welfare of older adults from several aspects. The details of this research process will be described subsequently in the remainder of this paper.

## 3. Methodology

In this section, the methodology for the prototype development, questionnaire survey, and interviews adopted in this study for designing and evaluating the desired interaction device for older adults are described. Two different types of interviews were conducted, one with the older users of the proposed device and the other with five invited experts in the related fields.

### 3.1. Prototype Development

In this study, by following the general guidance of prototype development mentioned in References [45,46,47], a prototype of the interactive device was constructed via the following steps: (1) establishing the principles for prototype design; (2) conducting a field experiment with users (the seven older adults in the previously-mentioned older people care center) being invited to use the prototype; (3) interviewing the users to collect their feedback and opinions about the usability of the prototype; (4) improving the prototype according to their feedback; and (5) testing the improved version in a second field experiment for future studies.

### 3.2. Questionnaire Survey

After modifying the prototype to obtain an improved version of the desirable interactive device as mentioned previously, 30 older adults in the older people care center were invited to test the effectiveness of the device in a second field experiment. After each participant finished using the device, a questionnaire survey of the participant’s opinions was conducted. The questions on the questionnaire were designed to be simple and concise, avoiding overly guiding the respondent, so that the data could be effective [48]. The questionnaire was divided into two parts: the first was based on the system usability scale (SUS) created by Brooke [49] with the questions being designed to be about the usability of the proposed interactive device and the answers based on a 5-point Likert scale [50]. In the second part of the survey, the questionnaire for user interaction satisfaction (QUIS) proposed by the Human-Computer Interaction Lab (HCIL) at the University of Maryland [51] was adopted. The questions, with answers also based on a 5-point Likert scale, were about the participating older adults’ feelings and satisfaction regarding their interaction with the interactive device prototype. The contents of the questionnaires will be described in detail later in Section 5.

### 3.3. Interview with the Older Users

In this study, interviews with the older adults in the aforementioned older people care center, who used the proposed device, were conducted according to the interview criteria mentioned in Reference [52] which points out that it is necessary to rely on the interviewer’s ability to guide different responding interviewees to create more open interactions and collect more diversified information. The interview outline designed for the users included three aspects: (1) the user’s operation situation; (2) the user’s interactive feeling; and (3) the design principle of the interactive device. The detailed items used in the interviews will be described later in Section 5.

### 3.4. Interview with Experts

In this study, five experts were invited to participate in the second field experiment of this study. Their expertises are listed in Table 2, including cognitive psychology, internal medicine, care service for older adults, and user interface design, etc. They were interviewed by the researchers of this study to express opinions about four aspects: (1) operation of the device; (2) design of the device; (3) design of the interface; and (4) suggestion for future developments. The details of their opinions will be described later in Section 5.

## 4. System Construction

In this section, after introducing the concepts for designing the proposed interactive device, the architecture of the device is presented, followed by the description of an algorithm of the “Guliu Guliu” game implemented on the device and the intermediate graphic outcomes yielded by an example of running the algorithm.

### 4.1. Design Concepts and Performance Process of the Proposed Interactive Device

**Figure 3:**
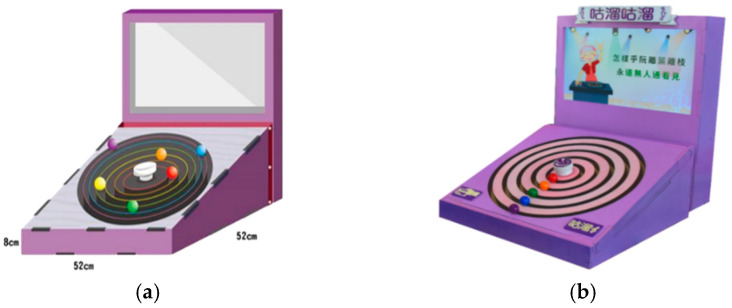
The proposed tangible interactive device. (**a**) An illustration of the design of the proposed interactive device. (**b**) A real construction of the device.

As shown in Figure 3, the interactive device proposed in this study was designed to simulate a music turntable of a nostalgic time. Seen from the exterior, it consists of a tangible panel and a display screen. At the center of the panel is a start button which may be pushed to start running the device. Around the push button are five concentric slide rails with five colored balls on them, each rail having one ball. The colors of the balls are red, yellow, green, blue, and purple in order from the center to the outer rail. An older user must slide dynamically the balls along respective rails, one at a time, according to the colors of the lyrics of a pre-selected song shown sequentially on the display screen to keep the melody of the song running continuously. The user performing this kind of game playing on the proposed device looks quite like a DJ operating a music mixer. Therefore, the device was named “Guliu Guliu,” whose pronunciation sounds like the noise made by an operating DJ mixer machine. An illustration of the device is shown in Figure 3a, and a real one constructed for this study is shown in Figure 3b.

The proposed device may be regarded as a nostalgic music machine on which an interactive game is played by an older person. It allows an older person to select and sing a favorite song of his/hers via the game. Meanwhile, he/she can stretch his/her hand muscles while rotating the concentric rails to keep the song selected by him/her running. This way of music playing and song singing not only brings entertainment to the older person in the interaction process but also increases the older person’s willingness to engage in the amusement activity.

In more detail, while a song is being played by the device and sung by an older user, the lyrics of the song are displayed sequentially on the display screen, each being in a random color. To keep running the melody of the song continuously, the user must respond by doing three tasks in a reasonable tempo for each lyric of the song:

(1) recognize the color of each lyric shown on the display screen;
(2) hold the corresponding colored ball on the panel promptly; and
(3) rotate the ball along the circular rail smoothly for a complete circle.

The first two of the above three steps must be completed within a fixed time duration. At the end of the above steps for all the lyrics of the song, the song will then be played completely for the older user to follow to sing.

With this method of interaction, the user can practice at least the following items of mind and body exercises:

(1) fast discrimination of colors by visual pattern recognition;
(2) quick hand movement to hold a color-correct ball by hand-eye synchronization;
(3) circular rotation of the ball along the slide rail; and
(4) singing a nostalgic song by recalling the lyrics in memory.

Afterwards, the device will give a score of the user’s performance according to the total response time he/she spent in the interaction process and keep it in a ranking record after he/she has completed the game.

In short, by using interactive device proposed in this study, it is expected to create the following effects:

(1) improving hand–eye coordination;
(2) boosting color recognition capabilities; and
(3) enhancing memory recall ability.

The songs chosen for use in this study are famous local melodies. They can be replaced to cater to the invited users’ hometowns or nationalities.

### 4.2. Architecture of the Proposed Interactive Device

**Figure 4:**
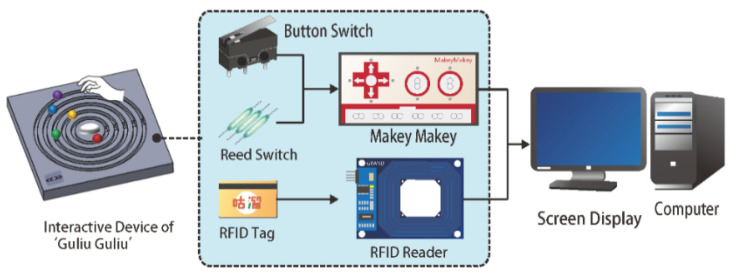
The Architecture of the proposed interactive device with a “Guliu Guliu” game.

The architecture of the proposed interactive device, as shown in Figure 4, is divided into two major parts. The first part is a software development platform that was built on a computer with a screen display and programmed using the software package of Adobe Flash for game controlling. The functions of the platform were presented previously in the last section. The second part is a hardware system with three components: a start button, five slide rails, and a radio frequency identification (RFID) reader.

As shown in Figure 3b, the start button is located at the center of the tangible panel and surrounded by the five concentric slide rails, and the RFID reader is located at an ID sensing area at the bottom left corner of the panel. A user may tap his/her user ID card, which includes an RFID tag, against the ID sensing area to trigger the RFID reader to read the tag and press the start button to turn on the button switch to generate a game-triggering signal. The signal is then sent, via the Makey Makey board, to the computer to start the “Guliu Guliu” game. Also, the user’s ID data are sent, in the meantime, to the computer for further processing and keeping in storage. It is noted, by the way, that the Makey Makey board is an invention kit through which everyday objects may be connected to a computer program [53]. Afterwards, when the user rotates the colored ball on one of the concentric slide rails to pass a fixed spot on the rail, the corresponding reed switch is turned on to generate a signal for use by the game program on the computer for song selections. Specifically, as mentioned previously, a rotation of the ball along the rail for a complete circle will pass the fixed spot two times to complete the selection of a song.

### 4.3. Algorithm of the “Guliu Guliu” Game

An algorithm named Algorithm 1 describing the details of the abovementioned process of playing the “Guliu Guliu” game is given in this section, followed by an illustrative example of the intermediate results yielded by running the algorithm in the next section. In addition, some steps in Algorithm 1 are explained additionally by comments written in smaller italic letters with a leading slash pair. They are added for better understanding of the involved logic in the algorithm.
**Algorithm 1.** The “Guliu Guliu” game performed on the proposed interactive device.**Input:** (1) a “waiting time” constant *T*_wait_; (2) three nostalgic songs and their lengths; (3) the trailers, the lyrics, and the corresponding music segments of three songs.**Output:** graphic pictures and videos shown on the display screen like those listed in Table 3.**Steps.*****//Stage 1: initialization ―*****Step 1:**                        *//Theme 1: “Game initialization” (inviting an action)* ask the user to press the start button to start the game by text and voice messages.**Step 2:**                            *//Theme 2: “Log in” (inviting an action)* ask the user to tap his/her ID card on the ID sensing area by text and voice messages.***//Stage 2: inviting the user to select a song ―*****Step 3:**  *//Illustrating how to select a song, inviting the user to do it, and playing the trailer of the selected song* 3.1teach the user how to select a song by rotating a ball with its color identical to that of thedesired-song title by displaying a text message and a sample song title;                             *//Theme 3: “Select a song” (teaching)* 3.2set the *initial time T*_o_ according to the system timer;        *//To is used for computing the total time used by the user to complete a round of the game* 3.3perform the following actions within a time duration of *T*_wait_ seconds: (a)ask the user by text and voice messages to select a song from the input three displayedon the screen as taught above in Step 3.1;     *//Theme 4: “Select a song” (inviting an action)* (b)**if** the selected song is *correct*, **then** *//Theme 5:* *“play the trailer of the selected song” (demonstration)*  (i)display a message to inform the user the selected song title and play the music trailer of the song;  (ii)go to Step 4;**else** go to Step 3.3 to ask the user to redo the selection; 3.4go to Step 1.      *//Restarting the game because the user fails to select a song within Twait seconds****//Stage 3: guiding the user to play the selected song one lyric after another ―*****Step 4:**    *//Illustrating how to play the song one lyric after another and inviting the user to do as illustrated* 4.1teach the user how to play the selected song one lyric after another in the following way viatext and voice messages as well as a short demonstration video:                           *//Theme 6: “Play a song lyric” (teaching)*  (a)select a ball with its color identical to that of each lyric displayed on the screen;  (b)rotate the ball for at least one circle around the rail; 4.2**for** each lyric of the selected song, **do**:                *//Theme 7: “Play all song lyrics” (inviting action and demonstration)*  (a)display the lyric with its text randomly assigned a color and ask the user to act astaught above in Step 4.1;  (b)**if** the user does *not* select a ball within a time duration of *T*_wait_ seconds, **then** go to   Step 1 to repeat the entire process;**end if;**  (c)**if** the selected ball is correct, **then**  **if** the ball is *not* rotated for a complete circle yet, **then** perform the following     steps repeatedly:     (i)display the lyric text with its characters highlighted one by one; and     (ii)play its music segment;          **end if;**        **else**          go to Step 4.2(a);**       end if**;**   end for**;***//Stage 4: ending ―*****Step 5:**                       *//Theme 8: Ranking of performances (illustration)* 5.1set the *ending time T*_e_ according to the system timer; 5.2compute the total game-running time as *T*_total_ = *T*_e_ − *T*_o_, normalize it as *T*_normal_ = *T*_total_*/T_k_* where*T_k_* is the length of the selected song; 5.3rank the user’s performance according to the value of *T*_normal_ based on the criterion: “theshorter the normalized game-running time, the higher the rank”; 5.4show the ranking result on the screen.**Step 6: while** the game is not restarted **do**:        *//Theme 9: Play the selected song (demonstration)*    play the entire melody of the selected song;                *//Playing repeatedly the entire selected song if the start button is not pressed*   **end while**;**Step 7:** go to Step 1.                    *//Starting the game from the beginning again*                                       *//End of the algorithm*

### 4.4. An Example of Results of Running the “Guliu Guliu” Game

An illustrative example of running the above algorithm is shown in Table 3, where the names of the themes mentioned in the comments of some intermediate steps in the algorithm are shown are shown in column 2, the outcomes (i.e., the graphic pictures shown on the display screen) of performing the themes are shown in column 3, their respective meanings are listed in column 4, and the corresponding step numbers are shown in the last column. The three possible outcomes of Theme 5 carried out by Step 3.3(b) are all shown in the table but only one of them will appear as the song selection result conducted by the user.

## 5. Results and Discussions

In this section, after presenting the details of the two field experiments conducted in this study, the analysis of the questionnaire data collected in the second experiment and the use of the data to evaluate the effectiveness of the proposed device are described, followed by the analysis of the collected users’ and experts’ interview comments for evaluating the effectiveness of the proposed device.

### 5.1. Design of the Field Experiment Process

The process for the field experiments conducted in this study was designed in the following way.

(1) Experimental place—the previously-mentioned older people care center in central Taiwan.
(2) Experimental subject—30 older adults, 65 years old or over with normal self-consciousness and not disabled.
(3) The time usage of the first field experiment for each participating older adult—the whole process took 30 min, including 5 min for a researcher of this study to explain the interaction process to the participant, 15 min for the participant to experience the interactions of the game, and 10 min for the researcher to conduct an interview with him/her.
(4) The time usage of the second field experiment for each participating older adult—the whole process takes 40 min, including 5 min to explain the interaction process to the participant, 15 min for the participant to experience the interactions, 10 min to do a questionnaire survey, and 10 min to conduct an interview with him/her.
(5) The experiencing process of the first field experiment—at the beginning, a researcher explained the interaction process to the participant; he/she started to experience the interactions after understanding the process; the researcher recorded the whole process by a video camera; and afterwards, an interview with the participant was conducted, aimed at understanding his/her feeling about the experiential process and general suggestions for modifications of the proposed prototype device for future uses.
(6) The experiencing process of the second field experiment using a modified version of the proposed device—the process was the same as that of the first field experiment except that the adopted device was an improved version of the originally designed one, and that before the interview, a questionnaire survey was conducted additionally, aimed at understanding more details about the participant’s feelings about the performing process with the improved interactive device. An evaluation of the proposed device was conducted afterwards.
(7) An interview with the experts—the experts mentioned previously were invited to participate in the second field experiment, observe the older adults playing the games in the entire experiment process, and be interviewed by the researchers of this study at the end of the experiment.

### 5.2. The Older Adults’ Experiencing Processes in the Two Field Experiments

#### 5.2.1. Records of the First Field Experiment

**Figure 5:**
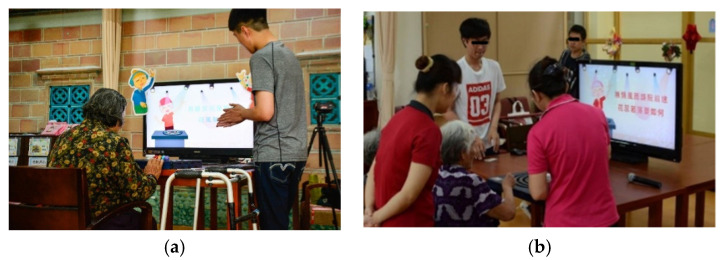
Scenes of the first field experiment. (**a**) A staff member of this study explaining the interaction process to a participating older adult. (**b**) Three staff members assisting a participating older adult to use the interactive device.

After the construction of the prototyping device with the “Guliu Guliu” game was completed, the first field experiment was conducted as described previously, followed by an evaluation of the opinions collected in the interviews with the participating older adults as well as a modification of the prototype device according to the evaluation results. A total of seven older adults were invited to participate in the experiment. Their average age was 81. Two scenes of the older adults using the interactive device are shown in Figure 5.

After each participant experienced the interaction process, a researcher of this study conducted an interview with him/her in which papers and pencils were used to record the participant’s responses rapidly. The questions asked in the interview were based on three aspects, namely, “user’s operation situation,” “user’s feeling about the interaction,” and “design principle of the interactive device.” The resulting records of the interviews were used to induce some general problems the older adults faced when using the proposed device. The problems included: the slide rail operation, the button design, the operation gesture, and some parts of the operation process that they could not understand on the display screen. It was also found in the interviews that the older adults thought that the singing activity was interesting, funny, and novel. According to these results, the prototype device was improved further for use in the next field experiment.

#### 5.2.2. Records of the Second Field Experiment

**Figure 6:**
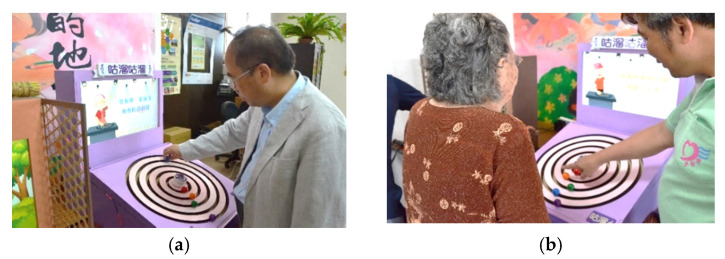
Scenes of the second field experiment. (**a**) A staff member of this study experiencing the operation of the interactive device. (**b**) A staff member of this study explaining the interaction process to a participating older adult.

After the prototype device was modified after the first field experiment, it was used in the second field experiment by more older adults to evaluate its effectiveness via questionnaire surveys and interviews. Two scenes of the second experiment are shown in Figure 6.

### 5.3. Analysis of Collected Questionnaire Data for Evaluation of the Effectiveness of the Proposed Device

In the second field experiment, after each older user had an interaction experience using the proposed device, a questionnaire survey of his/her opinions was collected by a researcher of this study. The total number of users responding to the survey was 30, and the number of valid questionnaires was 27. The content of the questionnaire was divided into three parts, namely, general information, system usability, and user interaction satisfaction, where the latter two parts were designed according to the concepts of SUS and QUIS, respectively, mentioned in Section 3.2. In this section, firstly, the collected data of each part were analyzed from the viewpoints of sample structure, reliability, and validity. Then, evaluations of the effectiveness of the proposed interactive device from the viewpoints of system usability and user interaction satisfaction were carried out.

#### 5.3.1. Analysis of the General Information of the Participating Older Adults

This first part of the questionnaire was used to collect the general information of the users of the proposed interactive device from four aspects, namely, age, living situation, exercise habit, and experience of using interactive devices. After a sample structure analysis of the collected data from these aspects, the following facts were found: (1) males accounted approximately for 26% and females for 74% of the users; (2) the average age of the users was 81 with approximately 11% being solitary older adults; (3) approximately 52% of the users indicated that they were not used to exercising regularly; and (4) approximately 89% of the users said that they had never used any interactive device before.

These statistics indicate that most of the older adults did not have habits of regular exercises and were not familiar with using interactive devices before.

#### 5.3.2. Adopted Methods for Analyzing Reliability and Validity of Collected Data

The *reliability* and *validity* of the collected data need be analyzed before the data can be used for evaluating the effectiveness of the proposed interactive device for older adults. Reliability is about the consistency of the data, despite repeated measurements [54]. In this study, the Cronbach’s alpha coefficient [55] was used for checking the consistency of a data set; and it is known that the closer the Cronbach’s alpha coefficient is to 1.0, the greater the internal consistency of the data. When the Cronbach’s alpha coefficient value is computed to be larger than 0.8, it is decided in general that the collected data are consistent and so may be regarded as reliable [56].

Validity is about the accuracy of a set of collected data, meaning that the data really express the concepts under consideration [54]. In this study, two methods were adopted for measuring the accuracy of a data set, namely, the Kaiser–Meyer–Olkin (KMO) measure of sampling adequacy and the Bartlett’s test of sphericity [57,58,59,60,61]. The KMO measure is a static with values lying between 0 and 1, indicating the proportion of variance among the data variables that might be caused by underlying factors, and a KMO measure value larger than 0.7 is thought generally to indicate that the accuracy of the collected data is acceptable. The Bartlett’s test of sphericity is used to test the hypothesis that the correlation matrix of the data variables is an identity matrix, and a significance level value smaller than 0.05 yielded by the test is thought generally to indicate that the collected data are accurate. When the results yielded by both methods indicate the accuracy of the collected data, the data in general are said to be valid. In addition, in this study the IBM SPSS (IBM, Armonk, NY, USA) package was used to analyze the collected data, while the Microsoft Excel application program was used for data tabulation.

#### 5.3.3. Analysis of Reliability and Validity of Collected Questionnaire Data

The questions in the second part of the collected questionnaire data about the *system usability* were divided into two aspects, namely, ease of use and applicability. The purpose was to investigate each participating older adult’s feeling about using the interactive device and the operational difficulty encountered while using the device. Ten questions were designed, as shown in the second column of Table 4, in which a half were positive questions and the other half are negative ones (marked by “P” and “N” in the table, respectively). A five-point Likert scale [59] was adopted to design the answers to the questions, i.e., the five choices of “strongly disagree,” “disagree,” …, “strongly agree” with scores 1, 2, …, 5, respectively, are provided as the scores of the possible answers to each question. The statistics of the percentages of *agreement* (including the scores of choices of “strongly agree” and “agree”) and the average scores of the answers to the questions are shown in the third and fourth columns of Table 4, respectively, which are a summary of Table A1 and Table A2 in Appendix A.

The analysis of the reliability and validity using the detailed data of Table A1 and Table A2 by the methods described in Section 5.3.2. using the SPSS software (IBM, Armonk, NY, USA) package was conducted in this study, resulting in the data shown in Table A3. It can be seen from the table that the computed Cronbach’s alpha coefficient was 0.901, which is larger than 0.8, indicating that the data were reliable. Also, the KMO measure was 0.768 which is larger than 0.7, and the significance level of the Bartlett’s sphericity test was 0.000 which is smaller than 0.05, both results indicating that the data were valid.

Furthermore, the questions of the third part of the questionnaire data about the *user interaction satisfaction* were divided into four aspects, namely, overall feeling, perceptual experience, learning situation, and connectivity. Sixteen questions were designed for these aspects, with four for each aspect, as shown in the second column of Table 5. The purpose was to investigate the user’s satisfaction about the interaction scheme and the components of the interactive device. Again, a five-point Likert scale [59] was adopted for use in the questions, where each question was designed to include two extremes of choices, like “boring/interesting” in the first question “I think the interaction style is (boring/interesting)” with “boring” corresponding to the score of “1” and “interesting” to the score of “5.” The statistics of the percentages of *agreement* (including the scores of the choices of “strongly agree” and “agree”) and the averages scores of the answers to the questions are shown in the third and fourth columns of Table 5 which is a summary of Table A4 and Table A5 in Appendix A. 

The analysis of the reliability and validity using the detailed data of Table A4 and Table A5 in the appendix was conducted as well, resulting in the data shown in Table A6. It can be seen from the table that the computed Cronbach’s alpha coefficient is 0.928 which is larger than 0.8, indicating that the data are *reliable*. Also, the KMO measure was 0.768 which is larger than 0.7 and the significance level of the Bartlett’s sphericity test was 0.000 which is larger than 0.05, both indicating that the data are *valid*.

The above results of the reliability and validity analyses of system usability and user interaction satisfaction show that the collected questionnaire data can be used further for evaluations of the effectiveness of the proposed interactive device, as done next.

#### 5.3.4. Evaluation of the Effectiveness of the Proposed Device According to Questionnaire Data

##### Evaluation of the Effectiveness from the Aspect of System Usability

The statistics of the average scores of the collected questionnaire data about *system usability* are shown in Table 4. As can be seen from the fourth column of the table, the adjusted average scores of all the questions except question 1 were all above 4.0; especially, those of questions 4, 5, and 8 achieve 4.5 or over. These facts indicate that the system usability of the proposed interactive device was considered by the users to be sufficiently good in general. More specifically, three major observations can be made, as described in the following.

(1) The overall average score of the six questions (questions 1 through 6) about the aspect of ease to use was 4.31—meaning that the design of the proposed interactive device meets the older adults’ habits and needs with no necessity of repeated explanations and extra information for them, and that they did not feel it troublesome or difficult to play the game as indicated by the text descriptions of the six questions.
(2) The average score of the four questions (questions 7 through 10) about the aspect of applicability of the device was also high up to 4.26—showing that the functions of the device were integrated suitably for the older adults with few inconsistencies so that they were willing and confident to use the interaction device, as indicated by the text descriptions of the four questions.
(3) Question 1 with an adjusted score of 3.96 was the only exception to the majority of above 4.0—saying that the interactive device is a little bit complicated and that the device still can be improved to make it simpler and more intuitive for older adults to use.

Another way to evaluate the effectiveness of the proposed device in the aspect of system usability is to adopt the method proposed by Bangor [62], by which a score in the range of 0 to 100 (from the negative sense to the positive) may be computed to rank the system in concern into five categories, namely, A (80–100), B (70–80), C (60–70), D (50–60), and F (0–49). The way to calculate the scores is as follows: (1) subtract 1 from the score of each positive question; (2) subtract the score of each negative question from 5 to yield a new score; and (3) sum up all the new scores and multiply the total by 2.5 to get the desired score. According to Sauro [63], a score so computed and reaching 68 or higher (i.e., better than class C) is said to meet the average standard, and a score of 80 or over may be regarded as excellent (i.e., belonging to class A). By using the detailed data in Table A2, the computed score of system usability of the proposed device for playing the “Guliu Guliu” game is 82.22 which indicates that the device is considered *excellent* for uses by the participating older adults.

##### Evaluation of the Effectiveness from the Aspect of User Interaction Satisfaction

The statistics of the collected data about the aspect of *user interaction satisfaction* are shown in Table 5 in which the average scores listed in fourth column of all the questions except questions 9, 10, and 14 can be seen to be larger than 4.0, indicating that the older users thought their interactions with the proposed device in the “Guliu Guliu” game were generally satisfactory. Specifically, the average scores of the questions about the four aspects—overall feeling, perceptual experience, learning situation, and connectivity with life are all—were all larger than 4.0, implying that the users’ satisfaction of the proposed device came *evenly* from all of the four aspects. In more detail, five major observations can be made, as described in the following.

(1) The average score about the aspect of overall feeling was 4.55—saying that the older users considered the game on the proposed device to be interesting, smooth, attractive, and delightful as indicated by the text contents of the four questions (questions 1 through 4) of this aspect in Table 5.
(2) The average score about the aspect of perceptual experience was 4.35—saying that the color brilliance and richness of the game displayed on the screen of the proposed device can be well perceived by the older users by vision and hearing and that the device is helpful for training of the older adults’ coordination capabilities.
(3) The average score regarding one’s learning situation was 4.04—indicating that the older users thought that the voice prompt provided by the game was clear and felt comfortable when learning how to use the device.
(4) The average score about the aspect of connection to life was 4.10—implying that the older adults thought that the device was helpful to their lives and could promote their relationships with others, indicating that the device can boost older adults’ willingness to join public activities in their daily lives.
(5) The scores of questions 9, 10, and 14 were below 4.0—indicating that the information provided in the gaming process may be made easier to read, the teaching animation can be made clearer, and the device may be improved further to provide the older adults better feelings related to their life experiences.

#### 5.3.5. A Summary of the Evaluation of the Effectiveness of the Proposed Device According to Questionnaire Data

The above evaluations of the effectiveness of the proposed device from the aspects of system usability and user interaction satisfaction now can be combined to reach the following conclusive remarks:

(1) the functions of the proposed interactive device are complete;
(2) the interface of the interactive device is simple to operate and easy to learn;
(3) the participating older adults felt pleasant while playing the game;
(4) the color brilliance and richness of the game displayed on the screen of the proposed device can be well perceived by the older users; and
(5) the information shown on the graphics of the game may be reduced.

### 5.4. Analysis of Collected User Interview Comments for Evaluating the Effectiveness of the Proposed Device

After an older person experienced the interactive game “Guliu Guliu” in the second field experiment, in addition to the questionnaire survey mentioned above, a researcher of this study also conducted an interview with him/her. Twenty-seven older adults were interviewed, with an average age of 81. In each interview, seven questions of three aspects were asked, namely, user’s operation situation, user’s interactive feeling, and design principle of the interactive device, as shown in Table 6 in which the collected opinions from the participating older adults are also shown.

Accordingly, it can be seen that the older adults in general held positive reflections on the game. Specifically, the following conclusions can be drawn:

(1) the participating older adults felt happy when they interacted with the device;
(2) the older adults were interested in the adorable characters shown on the screen during the game and could understand the game design concept;
(3) the older adults were able to resonate with the classical songs they listened to;
(4) the older adults thought that the device was helpful for cognitive ability enhancement and physical training;
(5) the older adults sometimes mistakenly recognized the colors, and worried about forgetting how to play the game afterwards.

### 5.5. Analysis of Data Collected from Interviews with Experts

In the second field experiment, five experts were invited to observe the game-playing processes of the participating older adults, and at the end of the experiment, they were interviewed by the researchers of this study from four aspects: operation of the device; design of the device; design of the interface; and suggestion for future developments. Their opinions were collected and listed in Table 7.

Accordingly, the experts’ opinions are summarized as follows:

(1) it is a good idea to promote the older person’s willingness to conduct exercises by an interactive device;
(2) the older adults were more receptive to activities and objects related to their experiences and cognitions;
(3) older adults can train their cognition and motor capabilities via the use of the interactive device;
(4) the design of the interfaces and interaction steps may be made easier and simpler for the older adults to understand and learn;
(5) the game-playing guidance can be made more intuitive; and
(6) older adults with cognitive impairments will have difficulty to perform the device and have no feeling of amusement.

The above conclusions of the experts’ opinions about the proposed system including the interactive device and the associated “Guliu Guliu” game will be used for improving the system for further studies.

## 6. Conclusions

Introducing modern technology into older adults’ life environments and creating friendly and healthy effects for them has been an issue which many scholars are concerned with. Through the literature review in this study, a better understanding of older adults’ physical and mental hypofunctions and successful active aging processes has been obtained, from which usage requirements and design principles of an interaction device for use in the active aging process were induced. Based on these findings, a tangible device with an interactive game named “Guliu Guliu” was constructed, which was based on the design concepts of connectivity, stimulus, simplification, and operational hint. It was a device which, by playing nostalgic music, allows older adults to enjoy entertainment and increase exercise through the interactive technology. By use of this device, it was aimed at knowing situations in which older adults use media and technology and analyzing the needs and problems they face. It was also desired to study older adults’ perceptual interaction experiences by various methods of questionnaire surveys, interviews, and in-field observations. These goals were fulfilled in this study as reported in this paper.

Two field experiments using the proposed interactive device were conducted. The opinions obtained from the interviews with the older users of the device in the first experiment were used for improving the initially-designed prototype device. Specifically, the maneuvering and interfacing mechanisms of the device and the associated “Guliu Guliu” game were modified to be more acceptable by the older adults to increase their willingness to face technological devices in their lives environments. In the second field experiment, the opinions of older users were collected via questionnaire surveys, interviews, and in-field observations from various viewpoints of human-machine interactions concerning older people’s uses of technology products. These data have been analyzed to show their reliability and validity, and then evaluated to prove successfully the effectiveness of the proposed interactive device for older adults.

In conclusion, the older adults invited to use the proposed interactive device were found interested in playing the proposed “Guliu Guliu” game and holding positive attitudes on it after they experienced the interaction process. In more detail, the following facts have been found through the above-mentioned field experiments and research processes.

(1) The older adults became more willing to use technological machines after using the device—
  In this study, an interactive device with an associated game was successfully designed to offer older adults a tool for experiencing technology games. In the process of interaction with the proposed interactive device, it was shown that the older adults were interested in playing the game on the device. Through the interviews with them after game playing, it was also found that they liked to use the device repetitively afterwards and felt satisfied with the game process.
(2) Involving the older adults in the interactive game created positive effects of active aging—
  From the questionnaire surveys and interviews, it was found that most of the older adults gave positive values on the proposed interactive device. They expressed that they felt relaxed when using the device and that the game was funny and could boost their hand-moving and cognitive-thinking capabilities when they used the device. These comments show that the proposed interactive device is quite helpful for an older adult to live an active-aging life.
(3) The older adults were more receptive to technology products after experiencing the interactions on the proposed device—
  Because the operations of the device were new to the older adults, they felt worried initially about whether they could use the device. However, it was found in this study that if the interactive device designed for use by the older adults was related to things they were familiar with (like the nostalgic songs, the animations with older-person characters, etc.), they would be more receptive to it regardless of the designs of the interface and operations. For example, the choice of the three well-known classical songs in this study did have recalled each participating older adult’s memory and eliminated his/her anxiety of getting used to technology when facing a machine for the first time.
(4) Using the interactive device can train the older person’s cognitive ability and increase his/her body exercises, reaching the desirable effect of sensory stimulus and health promotion—
  Through the interviews and in-field observations in the experiments, it was discovered that the older adults were able to stimulate their visual perceptions by watching the game interfaces when they used the interactive device to play the game. And they also have exercised the muscles of their hands to reach the effect of eye-hand coordination.
(5) More studies of the design should be directed to considerations of improving the system for uses by older adults with impairments in hearing, vision, cognition, and hand movement—
  The operations of the device are related to hearing, vision, cognition, and hand movement so that older adults with impairments in these aspects will have difficulty to perform the device, and will have psychological stress and no feeling of amusement while playing the game. Therefore, more considerations to improve the system for uses by older adults with the above-mentioned impairments should be made.

In the future, with the research experiences obtained in this study, more interactive devices like the proposed one with games aiming at promoting other aspects of older adults’ welfare and active aging may be developed and evaluated. Furthermore, it is also worth trying to include more advanced techniques like virtual reality, augmented reality, and mixed reality into the designs of interactive devices for use by older adults, helping to create friendlier LOHAS environments for active aging. Finally, about the evaluation of the effectiveness of the proposed device, it may also be adopted to have a sample of older adults use the interface of the device for a long time period and look at whether, relative to a control group, there are improvements in their wellbeing, sensory competence, etc.

## Figures and Tables

**Table 1:** Measures for compensating age-related deficits in sensory and cognitive functions [39].

Vision	Hearing
1. Increase levels of ambient and task illumination	1. Increase stimulus intensity
2. Increase levels of luminance contrast	2. Control background noise
3. Minimize the need to perform “near” work (i.e., to perform work at short distances)	3. Avoid the need to detect and/or recognize high-frequency acoustic information
4. Chose text font sizes of at least 12 points in character height	4. Avoid long-term exposure to high levels of noise (i.e., 88 dB or greater) across the life span
5. Deploy lighting strategies that minimize the opportunity for disability glare effects	5. Avoid the need to spatially localize low-frequency sound sources
6. Minimize dependence upon peripheral vision	6. Enhance speech recognition through the use of semantically well-structured prose that is rich in context and redundant
7. Adopt marking strategies that enhance motion perception and/or speed estimation capabilities	7. Improve speech recognition by presenting the speech at a reasonable and consistent pace
8. Use larger color contrast steps when discrimination between short wavelength (blue, green) colors	8. Complement hearing aid development by proper training of users and compensatory adjustments based upon user feedback
9. Explore the use of computer-based image processing techniques for optimizing the legibility of spatial form	9. Combine computer systems capable of real-time signal processing with ubiquitous Internet infrastructures to provide anytime/anywhere assistive listening support

**Table 2:** List of the backgrounds of invited experts interviewed in this study.

No.	Organization	Occupation	Expertise
A	LOHAS service center	director	psychology of aging, care service for older adults
B	older-adult care center	social worker	activity design for older adults, care service for older adults
C	national university	distinguished professor	user interface design, cognitive psychology, aging-related researches
D	hospital	physician	general medical disease, older people medical disease
E	private university	psychologist	cognitive behavior therapy, neuropsychology, depression, and psychotherapy

**Table 3:** List of intermediate interaction results yielded by the “Guliu Guliu” game described by Algorithm 1.

Theme Number	Theme of Interaction Step	Graphic Pictures Shown on the Display Screen	Meaning of Interaction Step	Step Number in Algorithm 1
1	“Game initialization” (inviting an action)	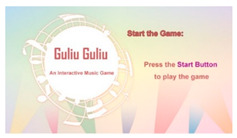	Inviting the user to press the start button to play the game.	Step 1
2	“Log in” (inviting an action)	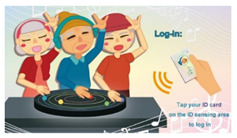	Inviting the user to tap his/her ID card on the ID sensing area.	Step 2
3	“Select a song” (teaching)	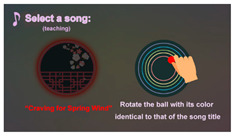	Teaching the user how to select a song by rotating the ball with its color identical to that of the song title.	Step 3.1
4	“Select a song” (inviting an action)	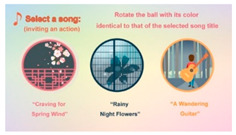	Inviting the user to select a song from three according to the teaching step (Step 3.1) illustrated above.	Step 3.3(a)
5a	“Play the trailer of the selected song” (demonstration) *(for song 1)*	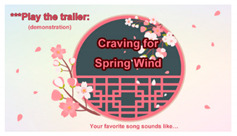	Playing the trailer of the song “Craving for Spring Wind” selected by the user before entering the game stage.	Step 3.3(b) *(for song 1)*
5b	“Play the trailer of the selected song” (demonstration) *(for song 2)*	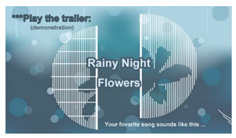	Playing the trailer of the song “Rainy Night Flowers” selected by the user before entering the game stage.	Step 3.3(b) *(for song 2)*
5c	“Play the trailer of the selected song” (demonstration) *(for song 3)*	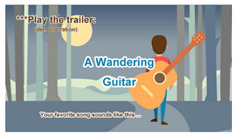	Playing the trailer of the song “A Wandering Guitar” selected by the user before entering the game stage.	Step 3.3(b) *(for song 3)*
6	“Play a song lyric” (teaching)	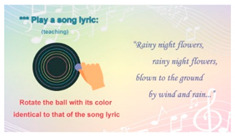	Teaching the user to play the song one lyric after another by rotating the ball with its color identical to that of the song lyric.	Step 4.1
7	“Play all song lyrics” (inviting an action and demonstration)	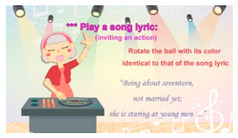	Inviting the user to rotate the ball with the same color as that of the lyric to play the music segments of the song lyrics one by another.	Step 4.2
8	“Ranking of performances” (illustration)	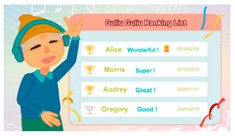	Displaying the ranking based on the data of the total time spent to play all the lyrics of a song in the game.	Step 5
9	“Play the selected song” (demonstration)	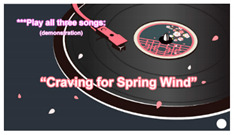	Playing the selected song at the end of the game for the user to enjoy or follow to sing together.	Step 6

**Table 4:** Questions about system usability in the questionnaire and statistics of percentages and averages of the scores of the answers to the questions.

Number	Question	% Agreement for Positive Question ^a^	Adjusted Average Score *
		or % Disagreement for Negative Question ^b^	
	A. Ease of Use	%	4.31
1	I think the interface of the interactive device is too complicated. (N) ***	77.77	3.96 **
2	I think it is easy to use the interactive device. (P) ***	100	4.41
3	I need repeated explanations to use the interactive device. (N)	81.48	4.04 **
4	I can learn to use the interactive device quickly like others. (P)	100	4.52
5	I think it troublesome to use the interactive device. (N)	100	4.63 **
6	I have to learn extra information to use the interactive device. (N)	96.30	4.30 **
	**B. Applicability**	**%**	**4.26**
7	I am willing to use interactive device often. (P)	91.48	4.15
8	I think the functions of the interactive device are integrated well. (P)	100	4.59
9	I think too many inconsistencies exist in the device interface. (N)	91.48	4.04 **
10	I have enough confidence to use interactive device. (P)	85.18	4.26

**^a^** % agreement = % strongly agree + % agree, **^b^** % disagreement = % strongly disagree + % disagree, * Adjusted average score for negative question = reversed average score = 6—average score of negative question according to Equation (A2) in Appendix A. ** Adjusted average score of negative question (average scores of positive questions are not adjusted). *** N: negative question, P: positive question. Underlines emphasize the different computations of the numbers.

**Table 5:** Questions about user interaction satisfaction usability in the questionnaire and statistics of percentages and averages of the scores of the answers to the questions.

No.	Question	% of Agreement for Positive Question ^a^	Average Score
	**A. Overall Feeling**	**%**	**4.55**
1	I think the interaction style is (boring/interesting).	96.30	4.59
2	I think the interactive device is (slow/smooth).	96.30	4.78
3	I think the interactive device is (dreadful/attractive).	88.89	4.33
4	I feel (frustrated/delightful) after using the interactive device.	92.60	4.48
	**B. Perceptual Experience**	**%**	**4.35**
5	I think the color of the game screen is (plain/brilliant).	100.00	4.70
6	I think the visual perception of the game screen is (monotonous/rich).	96.30	4.30
7	I think the hearing perception of the game screen is (monotonous/rich).	85.18	4.07
8	I think the device is (useless/helpful) to coordination training.	96.30	4.33
	**C. Learning Situation**	**%**	**4.04**
9	I feel (hard/easy) to read information in the screen.	66.67	3.70
10	I feel (confused/clear) about the teaching animation in the game.	59.26	3.59
11	I feel (confused/clear) about the voice prompt in the game.	85.29	4.15
12	I feel (stressed/comfortable) when learning how to use the device.	96.30	4.70
	**D. Connectivity with Life**	**%**	**4.10**
13	The device is (useless/helpful) to my life.	96.30	4.37
14	The device is (unrelated/related) to my life experience.	66.67	3.67
15	I think my relationship between people becomes (difficult/active) after using the device.	92.60	4.15
16	I think it is (useless/helpful) to boost people’s willingness to join activities after using the device.	96.30	4.22

**^a^** % agreement = % strongly agree + % agree.

**Table 6:** List of the questions used in interviews with the older adults using the proposed system.

Indicator of the Interview	Question of the Interview	User’s Response
User’s operation situation	1. Do you know how to play or operate this interactive device?	1. I know how to play but worry forgetting the way afterwards. 2. I know the location of the ID sensing area.
	2. Is there any part of the device that you don’t understand or you think need be improved when using this interactive device? For example?	1. The innermost rail is too close to the push button so that the rail ball cannot be slided smoothly, but this does not hinder normal operations of the game. 2. The operation is not difficult because the panel size is suitable for the hand to operate.
	3. Do you like the interface design of this interactive device? Why?	1. I like the design of the interface very much. 2. My eyes get tired while watching the TV but they won’t during game playing.
User’s interactive feeling	4. Do you feel happy when using this interactive device? Why?	1. The interaction is new and interesting. 2. The interaction enhances training of my cognitive capability, brain usage, and limb movement.
	5. Do you want to play it again? Why?	1. Yes, I will; and I like to play together with others. 2. I like to play my favorite songs repetitively. 3. I like to play as long as I have time.
	6. Do you have other ideas or feelings after you gain experiences from the interaction with the device?	I want to possess such a machine at home, but cannot because of insufficient space.
Design principle of the interactive device	7. Does it remind you of recalling old songs or other things after you gain experiences from the interaction with the device?	1. I like to sing and listen, and will recall my favorite songs like “Blue Melancholy,“ while interacting with the machine. 2. I don‘t think about the involved technology because of my limited academic background and knowledge.

**Table 7:** List of opinions collected from interviews with experts.

Indicator of Interview	Expert’s Response
Operation of the device	1. The ways of operations should be associated with the older person’s cognition in the brain. (B,C,E) * 2. It is a good idea to promote the older person’s willingness to conduct exercises by an interactive device. (A,D,E) 3. It has to use a bigger force to rotate the ball along the rail, so it is suggested to reduce the slide rail size so as to fit the arm mobility of older adults. (D) 4. For older adults who need rehabilitation, the size of the circular slide rail may be enlarged to let their hand joints move more. (E) 5. The time duration for playing the game should be no more than five minutes for older adults with cognitive impairments and may be longer than 15 min for healthy older adults. (D,E) 6. The operations of the device are related to hearing, vision, cognition, and hand movement so that older adults with impairments in these aspects will have difficulty to perform the device. (D,E)
Design of the device	1. The operations may be performed in a manner of “one purpose, one action,” like the linear operations on an ATM. (C) 2. Teaching of the operations may be conducted in a way of “Steps 1, 2, 3.” (C) 3. The device should be designed to be “easy to learn and operate.” (C,E) 4. The freedom of game playing may be increased. (C,D)
Design of the interface	1. The concept of “general use” may be adopted to design the interfaces. (C) 2. No error message is provided; this may be done by animation or voice prompts. (B) 3. The design of the colors of the balls on the slide rail should consider the usage by people with color blindness. (D,E) 4. Older adults with impairments of brain cognition, hand mobility, vision, and hearing using this device will have stress and no feeling of amusement. (D) 5. The device can be used to assist the older adults train hand-eye coordination, color recognition, and limb reaction. (E)
Suggestion for future developments	1. The collected data may be used for further applications. (C) 2. Melodies of other languages may be adopted. (A) 3. Training older adults to increase knowledge for using technology products may be enhanced. (B,C,D) 4. This device is useful for early detection of cognitive, hand-mobility, visual, and hearing impairments but not for diagnosis of these problems. (D) 5. It is suitable to increase the complexity of the design of the device (e.g., increasing the colors of the balls and the resistance of the slide rail) for uses by older adults who need rehabilitation. (E) 6. Older adults with impairments mentioned above will feel psychological pressure with no amusement effect while they are playing the proposed device. (D,E)

* Letters A through E in the parentheses denote the experts who gave the listed opinion.

## Appendix A

### Analyses of Reliability and Validity of Questionnaire Data about System Usability

The detailed statistics of the percentages of the scores of the answers to the questions of the second part of the collected questionnaire data about *system usability* are shown Table A1, and those of their averages are shown in Table A2.

The average scores of the questions listed in the fourth column in Table 4 are computed from the detailed data of Table A1 according to the following equation:

average score = 1 × (% of ‘strongly disagree’) + 2 × (% of ‘disagree’) + … + 5 × (% of ‘strongly  agree’). (A1)

Such average scores of the negative questions are underlined in the entries of the fourth column in Table 4 in Section 5.3.3. Their original values are listed in the entries of the fifth column in Table A2. Such original values are reversed into a *positive-sensed value* according to the following derivations before being filled into the entries of the sixth column of Table A2 and into the fourth column of Table 4 with the name of “adjusted average scores” for the convenience of an overall inspection of the positive effect of the questionnaire survey results:

reversed average score = 5 × (% of ‘strongly disagree’) + 4 × (% of ‘disagree’) + … + 1 × (% of ‘strongly agree’)        = (6 − 1) × (% of ‘strongly disagree’) + (6−2) × (% of ‘disagree’) + … + (6−5) × (% of ‘strongly agree’)                          = 6 − [1 × (% of ‘strongly disagree’) + 2 × (% of ‘disagree’) + … + 5 × (% of
‘strongly agree’)]                      = 6 − average score                    (A2)

where the last step is based on Equation (A1) above.

**Table A1:** Questions about system usability in the questionnaire and statistics of percentages of the scores of the answers to the questions.

No.	Question	Strongly Disagree	Disagree	Neutral	Agree	Strongly Agree
		1 *	2 *	3 *	4 *	5 *
	**A. Ease of Use**	(%) **				
1	I think the interface of the interactive device is too complicated. (N)***	25.92	51.85	14.82	7.41	0
2	I think it is easy to use the interactive device. (P) ***	0	0	0	59.26	40.74
3	I need repeated explanations to use the interactive device. (N)	29.63	51.85	11.11	7.41	0
4	I can learn to use the interactive device quickly like others. (P)	0	0	0	48.15	51.85
5	I think it troublesome to use the interactive device. (N)	62.96	37.04	0	0	0
6	I have to learn extra information to use the interactive device. (N)	37.04	59.26	0	3.70	0
	**B. Applicability**	(%) **				
7	I am willing to use interactive device often. (P)	0	3.70	14.82	44.44	37.04
8	I think the functions of the interactive device are integrated well. (P)	0	0	0	40.74	59.26
9	I think too many inconsistencies exist in the device interface. (N)	22.22	59.26	18.52	0	0
10	I have enough confidence to use interactive device. (P)	0	0	14.82	44.44	40.74

* Score values of 1–5. ** The values in the five columns are percentages (%). *** N: negative question, P: positive question.

**Table A2:** Questions about system usability in the questionnaire and statistics of the average scores of the answers to the questions.

No.	Questions	Min.	Max.	Average Score	Adjusted Average Score *	Standard Deviation
	**A. Ease of Use**				**4.31**	
1	I think the interface of the interactive device is too complicated. (N)	2	5	2.04	3.96 **	0.85
2	I think it is easy to use the interactive device. (P)	4	5	4.41	4.41	0.50
3	I need repeated explanations to use the interactive device. (N)	2	5	1.96	4.04 **	0.85
4	I can learn to use the interactive device quickly like others. (P)	4	5	4.52	4.52	0.51
5	I think it troublesome to use the interactive device. (N)	4	5	1.37	4.63 **	0.49
6	I have to learn extra information to use the interactive device. (N)	2	5	1.70	4.30 **	0.67
	**B. Applicability**				**4.26**	
7	I am willing to use interactive device often. (P)	2	5	4.15	4.15	0.82
8	I think the functions of the interactive device are integrated well. (P)	4	5	4.59	4.59	0.50
9	I think too many inconsistencies exist in the device interface. (N)	3	5	1.96	4.04 **	0.65
10	I have enough confidence to use interactive device. (P)	3	5	4.26	4.26	0.71

* Adjusted average score = reversed average score = 6 − average score of negative question according to Equation (A2) derived in the following. ** Adjusted average values of negative questions (average scores of positive questions are not adjusted). Underlines emphasize the different computations of the numbers.

For example, the average score of the first negative question in Table 4 about the aspect of ‘ease of use,’ namely, “I think the interface of the interactive device is too complicated,” is 2.04 which appears in the first entry in the fifth column of Table A2. It is transformed into the positive-sensed value of 3.96 in the following way according to Equation (A2) before being put into the first entry of the fourth column of Table 4 (also into the first entry of the sixth column of Table A2):

reversed average score = 6 − average score        = 6 − 2.04 = 3.96.  (A3)

The result of the analysis of the reliability and validity using the detailed data of Table A1 and Table A2 by the methods described in Section 5.3.2 using the SPSS software (IBM, Armonk, NY, USA) package is shown in Table A3.

**Table A3:** Analysis of reliability and validity of the collected questionnaire data about the system usability of the proposed interactive device.

Name of Measure or Test	Value
Cronbach’s alpha coefficient	0.901
Kaiser-Meyer-Olkin measure of sampling adequacy	0.768
Significance level of Bartlett test of sphericity	0.000

### Analyses of Reliability and Validity of Questionnaire Data about User Interaction Satisfaction

The detailed statistics of the percentages of the scores of the answers to the questions of the second part of the collected questionnaire data about *user interaction satisfaction* are shown Table A4, and those of their averages are shown in Table A5.

The result of the analysis of the reliability and validity using the detailed data of Table A4 and Table A5 by the methods described in Section 5.3.2 using the SPSS software (IBM, Armonk, NY, USA) package is shown in Table A6.

**Table A4:** Questions about user interaction satisfaction in the questionnaire and statistics of percentages of the scores of the answers to the questions.

No.	Question	Strongly Disagree	Disagree	Neutral	Agree	Strongly Agree
		1 *	2 *	3 *	4 *	5 *
	**A. Overall Feeling**	**(%)**				
1	I think the interaction style is (boring/interesting).	0	0	3.70	33.34	62.96
2	I think the interactive device is (slow/smooth).	0	0	3.70	14.82	81.48
3	I think the interactive device is (dreadful/attractive).	0	3.70	7.41	40.74	48.15
4	I feel (frustrated/delightful) after using the interactive device.	0	3.70	3.70	33.34	59.26
	**B. Perceptual Experience**	**(%)**				
5	I think the color of the game screen is (plain/brilliant).	0	0	0	29.63	70.37
6	I think the visual perception of the game screen is (monotonous/rich).	0	0	3.70	62.96	33.34
7	I think the hearing perception of the game screen is (monotonous/rich).	0	0	14.82	62.96	22.22
8	I think the device is (useless/helpful) to coordination training.	0	0	3.70	59.26	37.04
	**C. Learning Situation**	**(%)**				
9	I feel (hard/easy) to read information in the screen.	0	11.11	22.22	51.85	14.82
10	I feel (confused/clear) about the teaching animation in the game.	0	14.82	25.92	44.44	14.82
11	I feel (confused/clear) about the voice prompt in the game.	0	3.70	11.11	51.85	33.34
12	I feel (stressed/comfortable) when learning how to use the device.	0	0	3.70	22.22	74.08
	**D. Connectivity with Life**	**(%)**				
13	The device is (useless/helpful) to my life.	0	0	3.70	55.56	40.74
14	The device is (unrelated/related) to my life experience.	3.70	7.41	22.22	51.85	14.82
15	I think my relationship between people becomes (difficult/active) after using the device.	0	3.70	3.70	66.67	25.93
16	I think it is (useless/helpful) to boost people’s willingness to join activities after using the device.	0	3.70	0	66.67	29.63

* Score values of 1–5.

**Table A5:** Questions about user interaction satisfaction in the questionnaire and statistics of the average scores of the answers to the questions.

No.	Question	Min.	Max.	Average Score	Standard Deviation
	**A. Overall Feeling**			**4.55**	
1	I think this interaction style is (boring/interesting).	3	5	4.59	0.57
2	I think the interactive device is (slow/smooth).	3	5	4.78	0.51
3	I think the interactive device is (dreadful/attractive).	2	5	4.33	0.78
4	I feel (frustrated/delightful) after using the interactive device.	2	5	4.48	0.75
	**B. Perceptual Experience**			**4.35**	
5	I think the color of the game screen is (plain/brilliant).	4	5	4.70	0.47
6	I think the visual perception of the game screen is (monotonous/rich).	3	5	4.30	0.54
7	I think the hearing perception of the game screen is (monotonous/rich).	3	5	4.07	0.62
8	I think the device is (useless/helpful) to coordination training.	3	5	4.33	0.56
	**C. Learning Situation**			**4.04**	
9	I feel (hard/easy) to read information in the screen.	2	5	3.70	0.87
10	I feel (confused/clear) about the teaching animation in the game.	2	5	3.59	0.93
11	I feel (confused/clear) about the voice prompt in the game.	2	5	4.15	0.77
12	I feel (stressed/comfortable) when learning how to use the device.	3	5	4.70	0.54
	**D. Connectivity with Life**			**4.10**	
13	The device is (useless/helpful) to my life.	3	5	4.37	0.57
14	The device is (unrelated/related) to my life experience.	1	5	3.67	0.96
15	I think my relationship between people becomes (difficult/active) after using the device.	2	5	4.15	0.66
16	I think it is (useless/helpful) to boost people’s willingness to join activities after using the device.	2	5	4.22	0.64

**Table A6:** Analysis of reliability and validity of the collected questionnaire data about the user interaction satisfaction of using the interactive device.

Name of Measure or Test	Value
Cronbach’s alpha coefficient	0.928
Kaiser-Meyer-Olkin measure of sampling adequacy	0.768
Significance level of Bartlett test of sphericity	0.000
